# Quantitative protein expression profiling reveals extensive post-transcriptional regulation and post-translational modifications in schizont-stage malaria parasites

**DOI:** 10.1186/gb-2008-9-12-r177

**Published:** 2008-12-17

**Authors:** Bernardo J Foth, Neng Zhang, Sachel Mok, Peter R Preiser, Zbynek Bozdech

**Affiliations:** 1 School of Biological Sciences, Nanyang Technological University, Nanyang Drive, 637551 Singapore

## Abstract

**Background:**

Malaria is a one of the most important infectious diseases and is caused by parasitic protozoa of the genus *Plasmodium*. Previously, quantitative characterization of the *P. falciparum *transcriptome demonstrated that the strictly controlled progression of these parasites through their intra-erythrocytic developmental cycle is accompanied by a continuous cascade of gene expression. Although such analyses have proven immensely useful, the correlations between abundance of transcripts and their cognate proteins remain poorly characterized.

**Results:**

Here, we present a quantitative time-course analysis of relative protein abundance for schizont-stage parasites (34 to 46 hours after invasion) based on two-dimensional differential gel electrophoresis of protein samples labeled with fluorescent dyes. For this purpose we analyzed parasite samples taken at 4-hour intervals from a tightly synchronized culture and established more than 500 individual protein abundance profiles with high temporal resolution and quantitative reproducibility. Approximately half of all profiles exhibit a significant change in abundance and 12% display an expression peak during the observed 12-hour time interval. Intriguingly, identification of 54 protein spots by mass spectrometry revealed that 58% of the corresponding proteins - including actin-I, enolase, eukaryotic initiation factor (eIF)4A, eIF5A, and several heat shock proteins - are represented by more than one isoform, presumably caused by post-translational modifications, with the various isoforms of a given protein frequently showing different expression patterns. Furthermore, comparisons with transcriptome data generated from the same parasite samples reveal evidence of significant post-transcriptional gene expression regulation.

**Conclusions:**

Together, our data indicate that both post-transcriptional and post-translational events are widespread and of presumably great biological significance during the intra-erythrocytic development of *P. falciparum*.

## Abstract 2

A quantitative time-course analysis of protein abundance for Plasmodium falciparum schizonts using two-dimensional differential gel electrophoresis reveals significant post-transcriptional regulation.

## Background

Malaria is a serious parasitic disease that causes millions of deaths and incalculable suffering each year. It is caused by unicellular parasites of the genus *Plasmodium *that are transmitted between humans by a mosquito vector. A total of five species of *Plasmodium *parasites reportedly affect humans [1], with *P. falciparum *being by far the deadliest. *Plasmodium *parasites are characterized by a complex life cycle, during which they undergo extensive morphological and metabolic changes that reflect a robust adaptation of these parasites to the various host environments and ensure their growth and transmission.

After the injection of infectious sporozoites into the human host and an initial round of hepatocyte infection, the parasites replicate within red blood cells, progressing through an intra-erythrocytic developmental cycle (IDC) that takes the parasites about 48 hours to complete. Based on morphological appearance, the IDC has been divided into three developmental stages: ring, trophozoite, and schizont. The invasion of a red blood cell by a free, extracellular merozoite leads to the formation of the ring stage that lasts until 16 to 24 hours post-invasion (HPI). After a period of feeding and growth, the parasite enters the trophozoite stage (about 16 to 32 HPI), during which DNA replication begins. After repeated nuclear divisions, daughter cells are produced within the schizonts (about 32 to 48 HPI), with the release of multiple free merozoites marking the end of the IDC. This rapid asexual multiplication during the *Plasmodium *IDC causes the trademark clinical symptoms of the disease, ranging from fever, muscle aches and anemia, to organ failure, coma and death. The abundance of the blood-stage parasites and their prolonged occurrence in the human host render the IDC an important target of available antimalaria chemotherapies, as well as new drug-based and vaccine-based intervention strategies that are being developed.

Recent studies in *P. falciparum *have shown that morphological and metabolic development during the IDC is accompanied by large-scale, tightly controlled changes in gene transcription [2,3]. According to these transcriptome analyses, the vast majority of genes exhibit a cyclical expression pattern as the parasites progress through the IDC with a single peak in transcript levels. This rolling gene expression cascade was likened to a 'just-in-time' manufacturing process, in which induction of any given gene occurs at the time (or just before) it is required and in which the transcript is translated into its cognate protein without (much) delay. There is a remarkable conservation and rigidity of the IDC transcriptional cascade among different strains and species of *Plasmodium*, and genes from the same cellular or metabolic pathways often share similar profiles of mRNA abundance, ensuring their efficient function in the context of life cycle development [4,5].

However, several other studies have indicated that for many *Plasmodium *genes post-transcriptional regulation also plays a significant role in the expression of their protein products [6-11]. LeRoch and colleagues [6] conducted large-scale comparisons of mRNA and protein levels across seven major developmental stages of the *P. falciparum *life cycle. Although moderately high correlations were observed between the transcriptome and proteome of each stage, a significant fraction of genes were found to exhibit a delay between the peak abundance of mRNA and protein. In addition, the authors were able to identify a few consensus motifs in the 5'-untranslated regions that correlated with the transcript-protein accumulation pattern and are potentially involved in post-transcriptional regulation during the IDC. Another study [7] demonstrated that up to 370 transcripts that are produced during the gametocyte stage are translationally repressed until gamete fertilization via DDX6 RNA helicase-containing complexes. Relieving translational repression may lead to apparent translational upregulation, because investigators [8,9] showed that treatment with antifolate drugs can reduce the otherwise detectable translational suppression of the protein target of these drugs. Aiming for a fully quantitative approach, the same authors utilized two-dimensional gel electrophoresis with metabolically labeled proteins to characterize protein abundance across the *P. falciparum *IDC [10]. Again, results of these analyses suggested a widespread occurrence of post-transcriptional regulation in *Plasmodium *parasites. Such post-transcriptional regulation may also explain several discrepancies between mRNA abundance profiles and the expected timing of protein activity for several members of the *P. falciparum *pentose phosphate and REDOX metabolic pathways [12,13].

Besides translational control of protein expression, post-translational modifications (PTMs) have also been shown to play a critical role in the regulation of protein activity during the *Plasmodium *life cycle. These include proteolytic cleavage [14-18], glycosylation [19,20], phosphorylation [21,22], myristoylation [23], acetylation [24,25], and ubiquitination [26,27]. For example, the importance of proteolytic cleavage and glycosylation was established for various surface antigens, many of which are involved in merozoite invasion [14-16,19]. However, cytoplasmic proteins may also undergo specific PTMs that affect their enzymatic activities and/or cellular functions. Kumar and coworkers [17] showed that two *P. falciparum *phosphatases (PP7 and PP2B) are proteolytically truncated, leaving the active core intact. Altough the phosphatase activity of the full-length protein is sensitive to calcium concentrations, the processed core exhibits constitutive activity insensitive to calcium. For *Plasmodium *enolase, an essential glycolytic enzyme, at least five post-translationally modified isoforms have been found. Subcellular fractionation revealed differential enrichment of the enolase isoforms in different cellular compartment/fractions, including cytosol, cytoskeleton, membranes, and nucleus [21].

Taken together, these data indicate that translational regulation and PTMs (along with transcription) play a significant role in the timing of protein activities during the extensive transformations associated with the *Plasmodium *life cycle. However, we still lack a more detailed overview of the extent of post-transcriptional gene regulation and PTMs during the IDC, largely because most relevant studies either focused on particular proteomes (prepared by cell fractionation, after drug treatment, or from nonerythrocytic life cycle stages), employed nonquantitative or semiquantitative techniques, or examined only very broadly defined parasite stages such as rings, trophozoites, and schizonts (see, for example, references [10,11,19,28-31]).

In this study we used two-dimensional differential gel electrophoresis (2D-DIGE) [32] in quantitative proteomics analyses to investigate the extent of post-transcriptional gene regulation and PTMs during the late section of the *P. falciparum *IDC. We demonstrate that this technique provides high reproducibility suitable for quantitative measurements of relative protein abundance from samples collected at short time intervals from a highly synchronous *P. falciparum *culture. Using this approach we assembled high-resolution protein abundance profiles (four samples taken at 34, 38, 42, and 46 HPI) for 623 individual proteins/protein isoforms across the schizont-stage development. Of these, we identified more than 50 parasite protein isoforms by tandem mass spectrometry (MS/MS) and compared their protein expression profiles with the corresponding transcript levels observed from the same cell samples. Our data reveal striking examples of translational gene regulation and many instances of proteins that occur in multiple isoforms that are probably due to PTMs and/or pre-translational events, such as alternative splicing or transcription initiation/termination. Intriguingly, some protein isoforms exhibit expression patterns that are clearly distinct from those of other isoforms representing the same protein. We thus confirm that post-transcriptional events are widespread and of presumably great biological significance for *Plasmodium*, and that they should not be disregarded if a comprehensive functional analysis of its proteins is to be achieved.

## Results

### Experimental design

**Figure 1:**
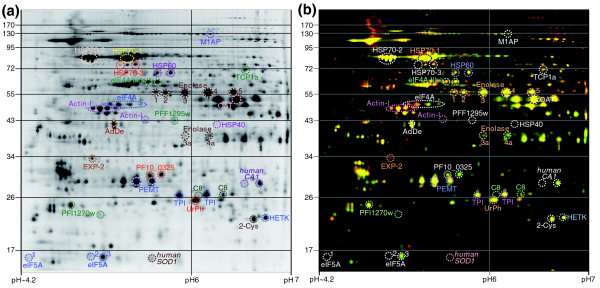
**Representative two-dimensional DIGE gels of *P. falciparum *schizont-stage proteins**. **(a) **Protein reference pool (internal standard) labeled with Cy2. **(b) **Overlay of images showing Cy3-labeled and Cy5-labeled parasite proteins from time point (TP) samples 1 (TP1, green) and 3 (TP3, red). Proteins were separated in the first dimension along a nonlinear pH gradient (pH3-7NL, 24 cm Immobiline DryStrip [GE Healthcare]), and in the second dimension on an 11% polyacrylamide gel. Proteins/protein isoforms identified by tandem mass spectrometry are highlighted in color. In instances where more than one spot was identified as the same protein, the spots were numbered in numerical order from left to right (not shown), except for enolase, for which spot numbers are denoted in the figure. The molecular weight marker is indicated in kDa. DIGE, differential gel electrophoresis.

**Figure 2:**
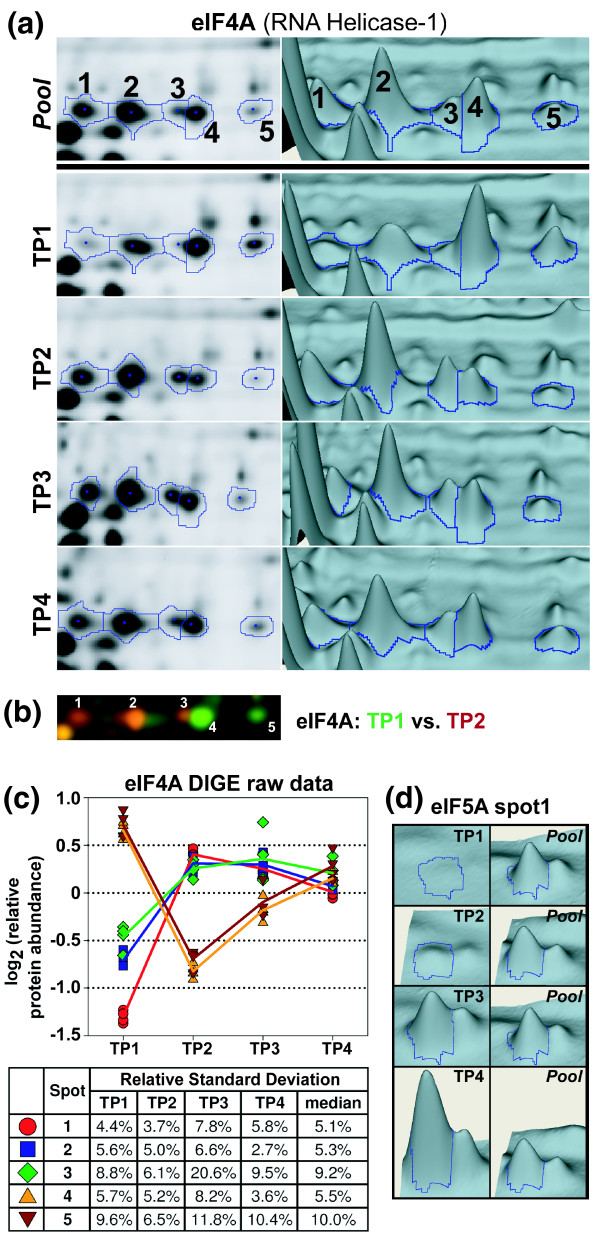
**Determining relative protein abundance using 2D-DIGE**. The relative protein abundance of a spot is defined as the normalized spot volume observed in the Cy3 or Cy5 channel (protein from a time point sample) divided by the normalized spot volume of the same spot measured in the Cy2 channel (protein reference pool) on the same gel. **(a) **gel images and three-dimensional 'landscape representation' of five protein spots identified as eukaryotic initiation factor (eIF)4A (or RNA helicase-1/helicase 45; PF14_0655) of *P. falciparum*. The top panel ('Pool') shows a representative image (gel3; see Table 1) of the Cy2-labeled protein reference pool/internal standard, whereas the lower panels depict one typical image for each of the four time point (TP) samples (TP1: Cy3/gel3; TP2: Cy5/gel7; TP3: Cy3/gel4; TP4: Cy5/gel6). **(b) **Overlay image of the Cy3-labeled and Cy5-labeled eIF4A isoforms from TP1 (green) and TP2 (red) from gel1. **(c) **Summary of the quantitative DIGE data and the resulting relative protein abundance profiles for the five eIF4A isoforms derived from all eight gels. The table presents the corresponding relative standard deviations for each set of four abundance measurements (for a given spot and time point sample) as well as the median value of the four relative standard deviations for each spot. **(d) **Three-dimensional presentation of eIF5A (PFL0210c) isoform 1, which happened to be the spot exhibiting the greatest fold change in the entire analysis (15.1-fold increase in relative protein abundance between TP1 and TP4). 2D-DIGE, two-dimensional differential gel electrophoresis.

2D-DIGE is a technique that allows quantitative measurements of relative abundance of individual proteins in complex samples [32]. Its key advantage is that - by using the three different fluorescent dyes Cy2, Cy3, and Cy5 - up to three different samples can be run simultaneously on one gel and quantitatively compared with one another. Here, we employed 2D-DIGE to measure relative protein abundance profiles in a time-course manner across schizont-stage parasites of *P. falciparum*. We collected parasite samples at 4-hour intervals at 34, 38, 42, and 46 HPI (referred to as time point [TP]1 to TP4). We also assembled a protein reference pool from the protein lysates of the four parasite samples that was labeled with Cy2 and used as internal standard throughout the entire study (Table 1). Each individual TP protein preparation was run in four separate experiments utilizing first-dimension strips spanning pH 3 to pH 7. To ensure the fidelity and unbiased character of the protein abundance measurements, each protein preparation was analyzed using both Cy3 and Cy5 flourophores in a dye-swap manner, and the sample loading scheme was designed such that the samples were assigned randomly to different gels (Table 1). The ratio between the fluorescence signals of the individual TP samples (Cy3 or Cy5) and the protein reference pool (Cy2) was used to assemble relative protein expression profiles (see below). Figure 1a shows a typical spot pattern of the protein reference pool resolved by two-dimensional gel electrophoresis (gel3, pH3-7NL, Cy2-channel), whereas Figure 1b shows the corresponding overlay image of the Cy3 (green) and Cy5 (red) signals derived from TP1 and TP3 samples, respectively. A total of 623 protein spots could be confidently discerned and matched across the eight gels, with the three-dimensional 'landscape representation' of the two-dimensional gel images generated by the DeCyder gel analysis software facilitating the matching and referencing of all protein spots across multiple gels (see Figure 2a). Often, more than one spot per gel was identified as the same protein (see below), and in such cases we use the term protein 'isoforms' to refer to the multiple protein products of the same gene. Such isoforms usually originate from PTMs such as phosphorylation, glycosylation, acetylation, acylation, ubiquination, or limited proteolysis (see, for example, reference [33]).

**Table 1:** Gel-loading regimen

Gel	TP1	TP2	TP3	TP4	Pool
gel1	Cy3	Cy5			Cy2
gel2	Cy5			Cy3	Cy2
gel3	Cy3		Cy5		Cy2
gel4	Cy5		Cy3		Cy2
gel5		Cy3	Cy5		Cy2
gel6		Cy3		Cy5	Cy2
gel7		Cy5		Cy3	Cy2
gel8			Cy3	Cy5	Cy2

Each two-dimensional gel was loaded with 50 μg of a Cy3-labeled time point (TP) sample, 50 μg of a different Cy5-labeled TP sample, and 50 μg of the Cy2-labeled internal standard (protein reference pool).

### Quantitative 2D-DIGE data

To arrive at relative protein abundance measurements using 2D-DIGE, we used the DeCyder software to calculate the raw background-subtracted volume for each protein spot and subsequently normalize these values (see Materials and methods, below, for details). For every spot, we calculated volume ratios that correspond to the ratio of the normalized spot volume from an individual protein sample (observed in the Cy3 or Cy5 channel) over the spot volume of the same spot from the protein reference pool (Cy2 channel). Given that this internal standard (Cy2) is identical in all gels, these volume ratios represent a reliable measure of a protein spot's relative abundance across multiple gels. In total, we included eight gels in the analysis that yielded 16 quantitative measurements for each spot (one observation in the Cy3 and one in the Cy5 channel of each gel). The average of the four measurements made for each spot per TP sample was thus used to establish the protein abundance profiles.

Figure 2 panels a to c illustrate this process for five protein spots that correspond to isoforms of RNA helicase-1, a protein that is also referred to as *P. falciparum *helicase 45 (PfH45) or as eukaryotic initiation factor (eIF)4A [34]. Interestingly, we observe two clearly distinct types of abundance profiles with three isoforms (1, 2, and 3), which initially increase in their intensity and level off for the rest of the time course, and two isoforms (4 and 5) that undergo a significant decrease through TP1 and TP2 and subsequent recovery in TP3 and TP4 (Figure 2c). These contrasting trends are also clearly visible in one gel in which samples from both TP1 (Cy3, green) and TP2 (Cy5, red) were run together (Figure 2b). The largest fold change in protein abundance of eIF4A was detected for isoform 1, which exhibited 3.3-fold increase between TP1 and TP2. In comparison the maximum fold change detected in the entire analysis was for one isoform of eIF5A, which exhibited a 15.1-fold increase throughout the time course (Figure 2d).

**Figure 3:**
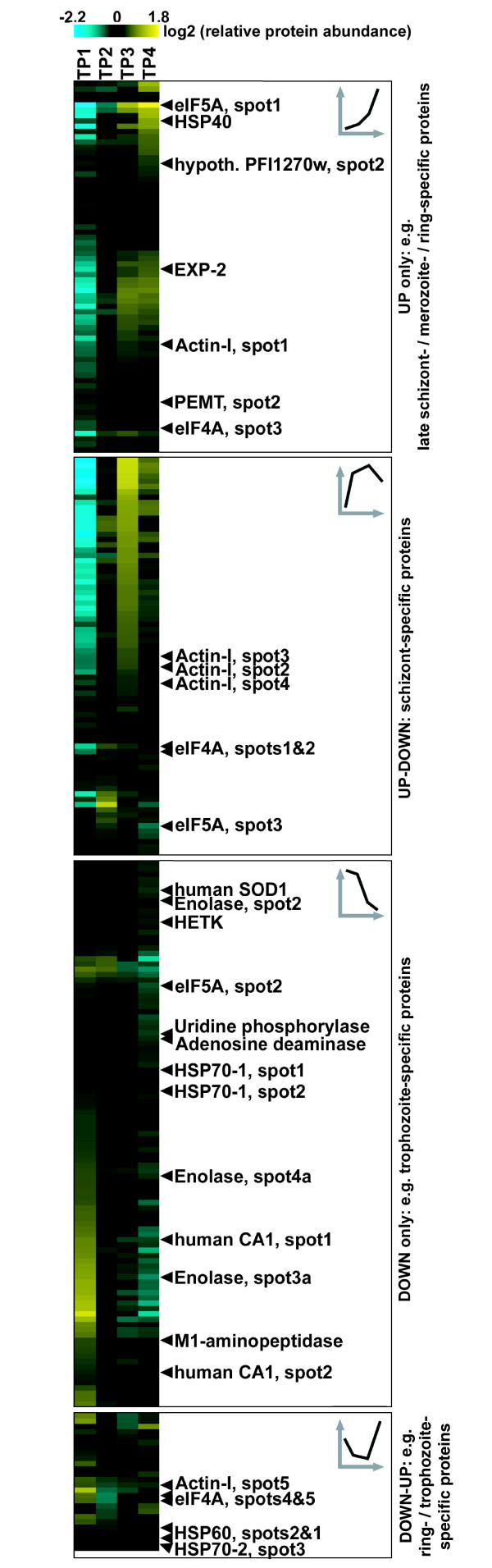
**Statistics of changes in relative protein abundance**. **(a) **Cumulative histogram of maximum fold change in relative abundance for proteins/isoforms that exhibit significant change (analysis of variance [ANOVA] *P *< 0.01) throughout the four schizont-stage time point (TP) samples. **(b) **the pie chart on the left illustrates how the 623 differential gel electrophoresis (DIGE) protein expression profiles are distributed among four categories defined by the statistical measures of variation (ANOVA), experimental reproducibility (median relative standard deviation [RelStDev]), and the maximum fold change (MFC) of relative protein abundance. The partial pie chart on the right provides an additional classification relating to the direction of abundance change, with the icons giving a generic illustration of each category.

To identify all proteins/isoforms whose abundance changes significantly through the schizont stage, we employed the one-way analysis of variance (ANOVA), as implemented in the DeCyder software. We find that a total of 345 proteins/isoforms exhibit abundance profiles with significantly (*P *< 0.01) greater variation in the measurements between the TP samples than within the TP samples. In addition, 278 of these proteins/isoforms also exhibit a fold change in excess of 1.4×, which - together with an ANOVA *P *< 0.01 - we chose as a criterion to delineate those proteins/isoforms whose change in abundance across the four TPs is more likely to be biologically significant. Of these, about one quarter (69 isoforms) change by more than threefold and 9% (24 isoforms) by more than fivefold (Figure 3a). Classifying these 278 expression profiles by the direction of their change (Figure 3b; see Materials and methods, below, for details), we find one-quarter (70 profiles) to increase steadily ('up'), approximately one-quarter (75 profiles) to exhibit an expression peak ('up-down'), and more than one-third (103 profiles) to decrease consistently ('down') during schizont development.

Unlike most studies that use 2D-DIGE to identify exclusively those proteins that are differentially expressed between different samples, we were also interested in the expression profiles of proteins/isoforms whose abundance did not change significantly (ANOVA, *P *> 0.01) across the four different TP samples. We therefore employed a second statistical measure of variation, the relative standard deviation (defined as standard deviation divided by arithmetic mean), to assess explicitly the reproducibility of protein abundance measurements. The relative standard deviation was calculated for each protein spot for each of the four TP samples, and the median of these four values was taken as a measure of experimental reproducibility for that spot (see Figure 2c and Table 2[35]). Comparison of these values with the graphical representation of the raw data (see Additional data file 1) illustrates the spread of the data. For the 278 proteins/isoforms that do exhibit significant abundance change across the four TP samples and a fold change in excess of 1.4×, the average value of their median relative standard deviations was 11.0%. Interestingly, this value also corresponds to the shoulder of the bimodal distribution of the median relative standard deviations of those 278 proteins/isoforms whose ANOVA result was nonsignificant (*P *> 0.01; data not shown). Using this value as a reproducibility threshold, we thus consider the abundance of 183 proteins/isoforms (29%) to exhibit minimal change through the schizont stage with high experimental confidence, thereby reflecting constitutive expression of these proteins/isoforms across the schizont stage. Another 96 proteins/isoforms (15%) that also do not show a significant change in expression do at the same time exhibit considerable experimental variation in the protein measurements (typically due to low signal levels; see Figure 3b).

**Table 2:** Protein data for the 54 protein isoforms identified in this study

Protein name	PlasmoDB ID and NCBI GenBank accession number	Calculated		Spot number	Mascot MS/MS ion search			Average volume ratio				Relative standard deviation					Maximum fold change	One-way ANOVA *P *value
								
		Mass (kDa)	pI		Score	Peptides matched	Sequence coverage	TP1	TP2	TP3	TP4	TP1	TP2	TP3	TP4	Median		
2-Cys peroxiredoxin	[PDB:PF14_0368] [Genbank:AAN36981]	22.0	6.7	-	559	4	48%	1.03	1.02	0.96	0.78	5.3%	15.4%	9.9%	6.4%	8.1%	1.33×	0.016

Actin-I	[PDB:PFL2215w] [Genbank:AAN36527]	42.1	5.2	1	533	9	40%	0.58	0.97	1.30	1.24	5.9%	5.9%	8.5%	3.2%	5.9%	2.24×	<0.001
				2	609	8	37%	0.60	0.93	1.44	1.25	4.8%	5.6%	5.6%	3.4%	5.2%	2.39×	<0.001
				3	391	8	36%	0.66	1.01	1.60	1.35	7.0%	4.4%	7.0%	2.2%	5.7%	2.43×	<0.001
				4	415	5	21%	0.63	0.94	1.21	0.97	7.7%	3.4%	8.6%	6.8%	7.3%	1.91×	<0.001
				5	192	6	29%	1.57	0.73	0.78	1.40	8.7%	7.0%	12.6%	11.3%	10.0%	2.15×	<0.001

Adenosine deaminase	[PDB:PF10_0289] [Genbank:AAN35486]	42.9	5.4	-	484	7	23%	1.24	1.18	1.15	0.80	14.5%	5.0%	2.6%	6.6%	5.8%	1.55×	<0.001

eIF4A/RNA helicase-1/helicase 45	[PDB:PF14_0655] [Genbank:AAN37268]	45.3	5.5	1	318	8	24%	0.41	1.32	1.19	1.01	4.4%	3.7%	7.8%	5.8%	5.1%	3.26×	<0.001
				2	159	2	10%	0.61	1.24	1.23	1.05	5.6%	5.0%	6.6%	2.7%	5.3%	2.03×	<0.001
				3	143	6	22%	0.73	1.20	1.30	1.16	8.8%	6.1%	20.6%	9.5%	9.2%	1.79×	<0.001
				4	133	4	19%	1.58	0.57	0.88	1.11	5.7%	5.2%	8.2%	3.6%	5.4%	2.78×	<0.001
				5	242	4	13%	1.64	0.62	0.94	1.22	9.6%	6.5%	11.8%	10.4%	10.0%	2.65×	<0.001

eIF4A-like helicase	[PDB:PFB0445c] [Genbank:AAC71878]	52.6	5.7	1	233	4	15%	0.99	1.05	1.24	1.22	7.9%	8.8%	15.7%	7.4%	8.3%	1.25×	0.024
				2	368	7	26%	1.02	1.06	1.18	1.13	7.8%	4.2%	7.6%	7.3%	7.4%	1.16×	0.045

eIF5A	[PDB:PFL0210c] [Genbank:AAN36131]	17.8	5.4	1	117	2	27%	0.12	0.32	1.26	1.79	18.2%	18.1%	10.9%	23.4%	18.2%	15.09×	<0.001
				2	129	2	16%	1.05	0.98	0.79	0.55	4.9%	12.3%	30.1%	28.8%	20.5%	1.9×	0.005
				3	151	2	16%	1.27	1.44	1.09	0.65	19.4%	24.0%	16.9%	21.6%	20.5%	2.23×	<0.001

Enolase	[PDB:PF10_0155] [Genbank:AAN35353]	48.6	6.2	1	344	10	30%	1.10	1.11	1.07	0.88	4.2%	4.4%	4.6%	7.2%	4.5%	1.26×	<0.001
				2	513	10	33%	1.15	1.18	0.99	0.83	14.1%	9.5%	4.8%	10.0%	9.8%	1.42×	<0.001
				3	627	10	33%	1.18	1.17	1.09	0.87	5.4%	5.0%	7.9%	8.5%	6.7%	1.35×	<0.001
				4	897	10	39%	1.13	1.06	1.11	0.92	11.7%	2.2%	8.3%	5.2%	6.7%	1.22×	0.016
				5	138	4	15%	1.03	0.91	1.14	0.93	19.9%	7.8%	9.9%	11.9%	10.9%	1.25×	0.125
				3a	213	5	14%	1.83	1.09	0.68	0.52	12.7%	12.9%	24.3%	3.3%	12.8%	3.52×	<0.001
				4a	122	3	11%	1.43	1.06	0.85	0.73	0.9%	3.1%	16.0%	4.8%	4.0%	1.94×	<0.001

EXP-2	[PDB:PF14_0678] [Genbank:AAN37291]	33.6	5.1	-	133	3	12%	0.27	0.70	0.88	0.97	8.1%	4.3%	10.4%	9.3%	8.7%	3.62×	<0.001

HSP40	[PDB:PFE0055c] [PDB:MAL5P1.12] [Genbank:CAD51377]	47.8	8.1	-	118	4	19%	0.70	0.85	0.94	2.06	13.2%	37.4%	14.2%	18.9%	16.5%	2.94×	<0.001

HSP60	[PDB:PF10_0153] [Genbank:AAN35351]	62.9	6.7	1	171	5	11%	1.29	0.96	1.10	1.37	8.6%	4.1%	7.0%	3.8%	5.5%	1.43×	<0.001
				2	235	5	11%	1.31	0.89	1.08	1.35	5.9%	3.8%	5.5%	6.5%	5.7%	1.51×	<0.001

HSP70-1^a^	[PDB:PF08_0054] [Genbank:CAD51185]	74.4	5.5	1	607	10	20%	1.31	1.13	1.01	0.93	14.7%	10.1%	12.9%	7.9%	11.5%	1.41×	0.008
				2	644	10	22%	1.46	1.23	1.06	0.96	5.7%	4.5%	10.8%	7.1%	6.4%	1.52×	<0.001

HSP70-2^a^	[PDB:PFI0875w] [Genbank:CAD51861]	72.5	5.2	1	486	8	17%	0.95	1.15	0.98	1.08	8.7%	5.0%	7.8%	8.1%	8.0%	1.2×	0.014
				2	724	10	23%	1.06	1.07	1.02	1.11	8.6%	4.8%	7.2%	4.4%	6.0%	1.08×	0.369
				3	694	10	21%	1.26	0.87	1.03	1.09	4.9%	2.5%	6.1%	4.1%	4.5%	1.46×	<0.001

HSP70-3^a^	[PDB:PF11_0351] [Genbank:AAN35935]	73.7	6.5	1	193	5	7%	1.08	0.97	0.97	1.06	7.2%	7.8%	16.2%	4.9%	7.5%	1.11×	0.299
				2	488	8	12%	1.01	0.98	1.16	1.11	8.0%	2.2%	11.3%	6.2%	7.1%	1.19×	0.028

CA1	- [Genbank:NP_001729]	28.9	6.6	1	264	5	32%	1.48	0.98	0.60	0.59	6.2%	4.3%	13.7%	3.8%	5.2%	2.5×	<0.001
				2	412	7	40%	1.16	0.94	0.72	0.77	6.0%	7.1%	17.4%	6.0%	6.6%	1.61×	<0.001

Human SOD1	- [Genbank:NP_000445]	16.2	5.7	-	247	5	57%	1.04	1.18	1.13	0.74	9.2%	12.5%	7.3%	16.2%	10.8%	1.6×	0.001

HETK	[PDB:PFF1335c] [PDB:MAL6P1.153] [Genbank:CAG25088]	20.6	7.0	-	575	8	56%	1.02	1.13	0.96	0.74	1.9%	9.0%	5.9%	7.5%	6.7%	1.53×	<0.001

Hypothetical protein PF10_0325	[PDB:PF10_0325] [Genbank:AAN35522]	33.2	5.6	1	380	7	33%	1.08	1.11	1.04	0.93	1.5%	7.0%	11.7%	14.8%	9.4%	1.2×	0.107
				2	497	8	33%	1.13	1.09	0.99	0.88	5.8%	4.9%	18.7%	17.9%	11.9%	1.28×	0.097

Hypothetical protein PFF1295w	[PDB:PFF1295w] [Genbank:CAG25080]	43.9	7.2	-	245	5	20%	1.06	1.04	0.95	0.91	2.2%	7.1%	13.7%	5.0%	6.0%	1.16×	0.072

Hypothetical protein PFI1270w	[PDB:PFI1270w] [Genbank:CAD51940]	24.9	5.5	1	332	6	21%	1.31	0.98	1.01	1.00	11.6%	8.7%	10.1%	14.3%	10.8%	1.34×	0.012
				2	74	3	17%	0.93	0.97	1.36	1.65	8.6%	16.8%	11.6%	9.8%	10.7%	1.77×	<0.001

M1AP	[PDB:MAL13P1.56] [Genbank:CAD52253]	126.6	7.3	-	171	7	10%	1.47	0.91	0.87	0.89	12.2%	8.7%	13.4%	10.9%	11.5%	1.69×	<0.001

OAT	[PDB:PFF0435w] [PDB:MAL6P1.91]	46.0	6.5	-	589	9	29%	1.20	1.05	1.06	1.06	0.9%	5.8%	12.0%	5.4%	5.6%	1.14×	0.116

PEMT	[PDB:MAL13P1.214] [Genbank:CAD52560]	31.3	5.4	1	790	9	46%	1.07	1.12	1.09	0.89	7.2%	10.3%	9.6%	16.7%	9.9%	1.26×	0.049
				2	182	3	14%	0.76	0.97	1.13	1.05	6.2%	5.8%	14.0%	16.3%	10.1%	1.49×	0.003

Proteasome component C8	[PDB:PFC0745c] [Genbank:CAB11152]	29.7	6.4	1	67	1	7%	1.17	1.20	1.12	0.83	12.9%	3.1%	5.1%	7.6%	6.4%	1.44×	<0.001
				2	275	4	28%	0.98	0.96	0.96	0.82	7.4%	9.2%	10.8%	11.2%	10.0%	1.2×	0.072

TCP1a	[PDB:PF11_0331] [Genbank:AAN35915]	60.6	6.7	-	110	5	11%	1.05	0.97	1.04	1.04	3.0%	4.1%	6.4%	6.2%	5.1%	1.08×	0.190

TPI	[PDB:PF14_0378] [Genbank:AAN36991]	28.1	6.0	1	579	8	43%	1.18	1.13	1.04	0.86	6.1%	4.5%	11.8%	9.4%	7.7%	1.37×	<0.001
				2	578	8	45%	1.20	1.11	1.07	0.88	7.0%	5.1%	10.0%	9.6%	8.3%	1.37×	<0.001

Uridine phosphorylase	[PDB:PFE0660c] [Genbank:CAD51497]	27.5	6.1	-	430	7	35%	1.23	1.16	1.09	0.73	5.2%	3.6%	7.0%	6.5%	5.8%	1.68×	<0.001

The average volume ratios represent log_2_-transformed values and have not been mean-centered around zero. ^a^Nomenclature in agreement with Shonhai and coworkers [35]. CA1, human carbonic anhydrase 1; eIF, eukaryotic initiation factor; EXP, exported protein; HETK, hydroxyethylthiazol kinase; HSP, heat shock protein; M1AP, M1-family aminopeptidase; OAT, ornithine aminotransferase; PEMT, phosphoethanolamine N-methyltransferase; SOD, superoxide dismutase; TCP1a, T-complex protein 1, α subunit; TPI, triose phosphate isomerase.

**Figure 4:**
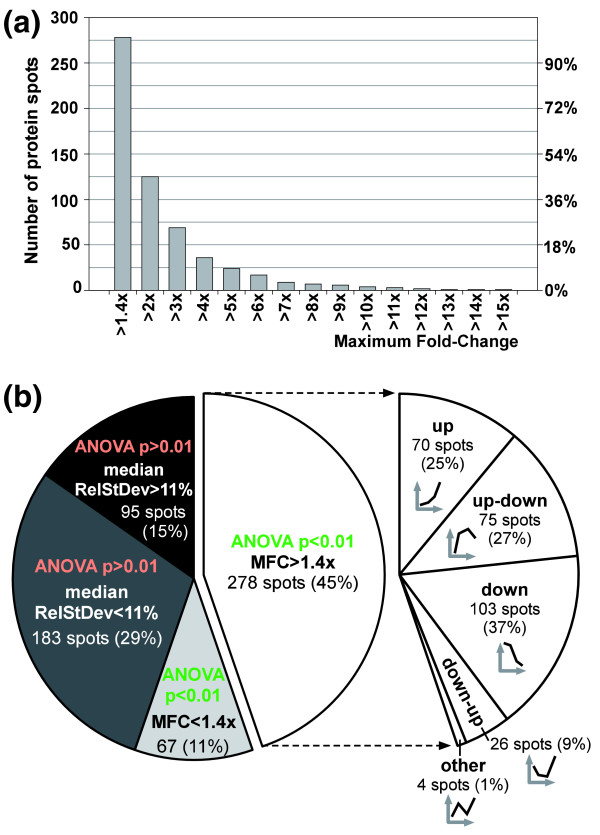
**Overview of relative protein abundance dynamics during the *P. falciparum *schizont stage**. The 278 differential gel electrophoresis (DIGE) protein expression profiles that exhibit a statistically significant change (analysis of variance [ANOVA] *P *< 0.01) and a considerable maximum fold change (>1.4×) across the *P. falciparum *schizont development were grouped according to the direction of abundance change (see Figure 3b) and subsequently subjected to hierarchical clustering, with the icons in the upper right corner of each panel providing a generic illustration of each category.

To create an overview of global protein abundance dynamics during the *P. falciparum *schizont stage, we carried out hierarchical clustering with the 278 protein abundance profiles that exhibit a significant ANOVA (*P *< 0.01) and a fold change in abundance in excess of 1.4× (Figure 4), with the four panels in Figure 4 corresponding to the categories already mentioned above ('up', 'up-down', 'down', and 'down-up') and indicated in Figure 3b. These data show, for example, that most isoforms of the invasion-related molecule actin-I exhibit an expression peak during late schizont development, as is expected based on its function [36]. Also, in many cases multiple isoforms of a given protein vary greatly from one another in their expression pattern (for example, eIF5A). A detailed analysis and discussion of protein abundance and function is presented below.

### Protein identification

A total of 54 protein spots were excised from two-dimensional gels and confidently identified by tandem mass spectrometry (MS/MS) and Mascot searching of the MS/MS data against GenBank's nr database as well as a custom database containing *Plasmodium *and human proteins. For almost all identified protein spots, Mascot matched three or more individual peptides yielding a sequence coverage of more than 10% and a Mascot score (probability-based Mowse score) that is considerably greater (score typically >100) than the significance threshold (ion score of 35 to 55 for *P *< 0.05) given by the software (Table 2). In addition, the positions of these proteins on our two-dimensional gels are in good agreement with calculated masses and pI values (Figure 1 and Table 2) as well as with previously published data [10,36]. The 54 identified protein spots were found to derive from a total of 24 parasite and two human proteins, with 15 of these proteins having been encountered in more than one protein spot (Figure 1 and Table 2). The limited scope of identified proteins notwithstanding, these findings suggest that more than 50% of *Plasmodium *proteins might be present in numerous isoforms in the cell, which are probabaly due to PTMs and/or alternative pretranslational events. Enolase represents the protein with the highest number of isoforms (7) detected in this study (Figure 1).

### Protein expression profiles and mRNA levels

**Figure 5:**
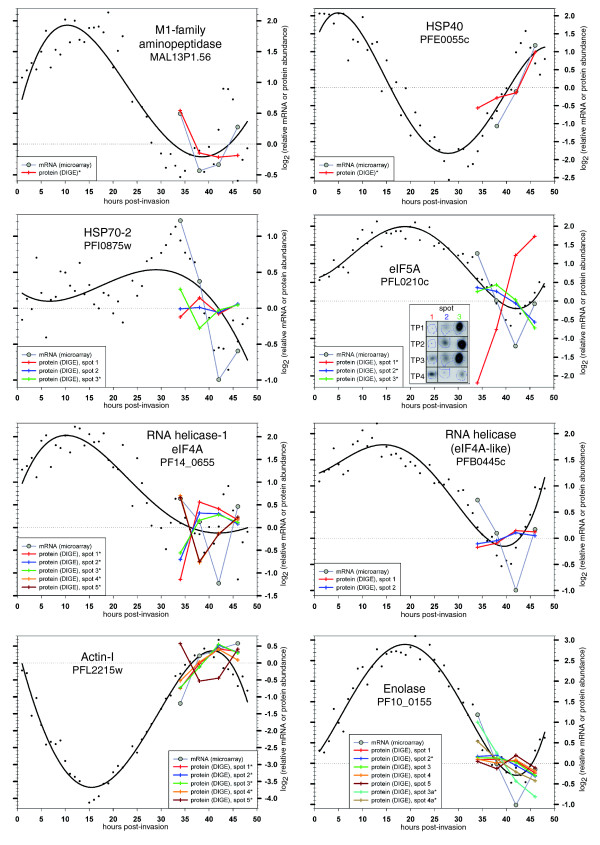
**Expression profiles comparing relative mRNA and protein abundance**. The panels summarize mRNA and protein abundance data for individual *P. falciparum *genes, with PlasmoDB accession numbers indicated below the gene names. Both the relative mRNA and protein abundance levels determined in this study for four schizont-stage time points (TPs) are based on the same parasite samples from a single large-scale *in vitro *culture and are indicated by gray and colored lines (see panel legends), respectively. In addition, the previously published transcript dynamics across the whole intra-erythrocytic life cycle [2] are included as black lines, with the black dots representing the corresponding raw data. All abundance profiles were mean-centered around zero based on the data points between 34 and 46 hours post-invasion. Asterisks in the panel legends denote cases for which analysis of variance (ANOVA) *P *< 0.01 and maximum fold change >1.4×. The insert in the eukaryotic initiation factor (eIF)5A panel depicts actual two-dimensional gel images to provide an approximate impression of absolute protein abundance levels between the three eIF5A isoforms.

For the parasite proteins identified by mass spectrometry, we then compared the DIGE protein expression profiles with the following: microarray data that we generated from the same parasite samples that were used for the proteomic analysis, and with the previously published *P. falciparum *IDC transcriptome [2]. The microarray data produced in this study are in good agreement with the high-resolution IDC transcriptome, confirming the tight synchronization and appropriate progression of our parasite culture through schizont development (Figure 5; see Additional data files 2 to 4). In addition, the comparisons of the 2D-DIGE data with the transcription data reveal that the expression profiles of some proteins/isoforms closely mirror their mRNA levels, for instance for M1-family aminopeptidase, heat shock protein (HSP)40, and all but one isoform of actin-I.

Intriguingly, in many other cases the protein expression levels appear to lag behind or to be decoupled from the corresponding mRNA levels (Figure 5). One of the most striking examples is eIF4A, for which three isoforms (spots 1 to 3) exhibit an expression peak around TP2/TP3 and are essentially anticorrelated to the corresponding mRNA levels, whereas two other isoforms of the same protein (spots 4 and 5) exhibit a sharp dip in expression at this time, thus somewhat resembling the changes in mRNA abundance (Figure 5). Similarly, two isoforms of eIF5A (spots 2 and 3) show a modest decrease in expression during schizont development and thereby mirror the mRNA levels with a delay, whereas one isoform with a considerably more acidic isoelectric point (spot 1) exhibits a 15-fold increase in expression (Figure 5). Another example is enolase, for which most full-length isoforms (spots 1 to 5) show only minor changes in protein abundance, whereas two isoforms that correspond to a lower molecular mass (spots 6 and 7) are characterized by a gradual decrease in expression throughout the time course and thus resemble the corresponding mRNA levels more closely (Figure 5). Finally, although the expression profiles of four actin-I isoforms (spots 1 to 4) are almost identical to their mRNA profiles, one apparently truncated isoform (spot 5; Figure 1) does exhibit an essentially anticorrelated profile (Figure 5). These examples illustrate that during the *P. falciparum *IDC each protein isoform exhibits a specific abundance profile that may dramatically differ from the corresponding mRNA profile and/or from the protein abundance profiles of other isoforms of the same protein. These findings are consistent with the notion that different isoforms of a given protein may serve different biological roles during the IDC of *Plasmodium *spp. [10,21].

### Western blot analyses

**Figure 6:**
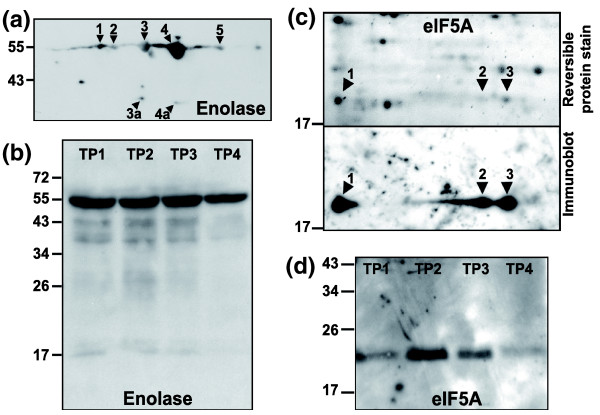
**Immunoblot analysis of enolase and eIF5A in *P. falciparum *schizont-stage parasites**. **(a) **Two-dimensional Western blot of enolase. **(b) **One-dimensional Western blot of enolase. **(c) **Two-dimensional Western blot of eukaryotic initiation factor 5A (eIF5A). The upper panel shows the blotted nitrocellulose membrane after staining with a reversible protein dye (MemCode; Pierce), whereas the lower panel depicts the resulting immunoblot after detection with antibodies raised against a plant eIF5A. **(d) **One-dimensional Western blot of eIF5A. For each two-dimensional blot, 500 μg parasite protein (time point [TP]3, 42 hours post-invasion) was separated on a 13 cm isoelectric focusing (IEF) strip pH 4 to 7 (GE Healthcare) followed by an 11% polyacrylamide gel. Protein spots that could be matched on silver-stained gels were confirmed as *P. falciparum *enolase (panel a) and eIF5A (panel c) by tandem mass spectrometry and are indicated by arrowheads. For the one-dimensional blots, each lane was loaded with 10 μg parasite protein from the corresponding time point sample. eIF, eukaryotic initiation factor.

In order to validate the protein spot identifications made by mass spectrometry and the 2D-DIGE protein abundance measurements, we conducted Western blot analyses focusing on two *P. falciparum *proteins, namely enolase and eIF5A (Figure 6). Antibodies raised against the full-length *P. falciparum *enolase [37] recognized at least 10 protein spots on the two-dimensional Western blot (Figure 6a). Seven of these spots that could be matched on silver-stained gels (spots 1 to 5, 3a, and 4a) were also analyzed by mass spectrometry and confirmed as enolase (Table 2). The one-dimensional blot (Figure 6b) revealed that the expression level of both full-length enolase (about 55 kDa) and of several much fainter bands at lower molecular weight (about 35 to 45 kDa) remained more or less constant through TP1 to TP3, and decreased somewhat in TP4, which is in good agreement with the expression profiles yielded by 2D-DIGE (Figure 5).

For eIF5A, we used antibodies that had originally been raised against the eIF5A protein from tobacco plants [38] but had also been used successfully to detect this protein in *P. vivax *[39]. These antibodies detected three protein spots on the two-dimensional Western blot (run with protein from TP3) that migrate at approximately 18 kDa, which corresponds to the predicted molecular weight of *P. falciparum *eIF5A (Figure 6c, lower panel). All three spots were also excised from silver-stained two-dimensional gels and identified as *P. falciparum *eIF5A by mass spectroscopy (Table 2). Similar to enolase, we observe a good correlation between the protein abundance profiles detected by 2D-DIGE and Western blotting. The total protein abundance profile revealed by the one-dimensional Western blot is characterized by a slight increase from TP1 to TP2 and the rapid decline through TP3 and TP4 (Figure 6d). This pattern mirrors the DIGE expression profiles of spots 2 and 3 (Figure 5), whereas the differentially expressed spot 1 - because of its low absolute expression level (see inset in Figure 5) - presumably does not contribute significantly to the overall changes in protein abundance.

## Discussion

### Protein expression profiles

2D-DIGE is a powerful quantitative proteomics technique [32,40] that is commonly employed to study cells or tissues under two or more experimental conditions, but it is rarely used to uncover large-scale proteome changes during the natural development of biological systems (see, for example, reference [41]). In this study we show that 2D-DIGE is well suited to generate time-course protein expression profiles in a medium-throughput manner for malaria parasites as they progress through their IDC.

Previous investigations of large-scale quantitative protein changes in *P. falciparum *considered very broadly defined parasite stages and, in particular, divided the approximately 48-hour IDC into three phases (rings, trophozoites, and schizonts) [10,28,29]. In contrast, the proteome time course experiment presented here is based on four TP samples taken at 4-hour time intervals, with the resulting protein expression profiles revealing proteome changes in schizont-stage parasites at the highest temporal resolution ever attempted. Furthermore, although some of the previous studies employed semiquantitative mass spectrometric measures to quantify protein abundance [28,29], this report is - to the best of our knowledge - the first to make use of the exquisitely quantitative 2D-DIGE technology [32,40] in malaria parasites. For the 623 proteins/isoforms analyzed in this study we generated a total of more than 9,000 individual protein abundance measurements (for four TPs and taken in quadruplicate per spot). The high resolution and reproducibility of this approach thus allow for in-depth investigation into protein (isoform) dynamics in the malaria parasite.

The overall biological relevance of the expression profiles is indicated by the fact that they exhibit trends that one would plausibly expect to observe in schizont-stage malaria parasites. More than half of all protein spots exhibit a statistically significant change in protein abundance across the four TPs, which is consistent with the fact that most transcripts show a wave-like expression pattern during the IDC [2]. Of the 278 proteins that also show a considerable fold change (>1.4×) in abundance, approximately one-quarter exhibit an expression peak during schizont development. In particular, closer examination of the expression profiles of actin-I, a protein that is part of the molecular motor machinery essential for erythrocyte invasion (which occurs between schizont and ring stages) and whose expression is expected to be significantly upregulated in schizonts [42], confirms a significant increase in DIGE-measured expression from TP1 to TP3 (Figure 5).

### Comparing transcript and protein abundance

The direct comparisons of relative levels of transcript and protein expression, both determined from the same parasite preparations, yielded valuable insights into their different possible correlations. In many cases we find that changes in transcript levels are clearly mirrored by corresponding changes in protein abundance, for example in HSP40 and most isoforms of actin-I (see Figure 5). In both cases transcript and protein levels increase, suggesting that here an increased number of transcripts leads directly to a higher volume of translation and protein abundance.

In many other instances, though, changes in mRNA and protein levels are less well correlated, which may reflect sequence-specific factors and post-transcriptional regulation affecting both protein synthesis and/or degradation [43,44]. In yeast, translation rates vary greatly between transcripts, and the underlying molecular mechanisms probably include codon usage, transcript length, saturation effects, ribosomal occupancy, and translational suppression [45-48]. On the other hand, the major pathway of targeted protein degradation - ubiquitin-mediated proteasome activity - has already been shown to be present in *P. falciparum*, to be developmentally regulated, and to be essential for intra-erythrocytic development [26,49-51]. We find that for some proteins changes in protein abundance parallel changes in transcript levels after a delay; examples include isoforms 2 and 3 of eIF5A, adenosine deaminase, hydroxyethylthiazole kinase, proteasome component C8, and uridine phosphorylase (see Figure 5 and Additional data file 2). In these examples the transcript and protein levels both decrease, and the time delay could be explained by an initially low protein turnover rate. As soon as the transcript levels drop and corresponding protein translation decreases, the proteins already present in the cell remain stable for some time before being degraded. Alternatively, it could also be that the maximum translation rate for a given protein is already reached at a less-than-maximum transcript concentration, with the translation rate dropping only once the transcript level falls below a certain threshold. Other cases show a decrease in transcript levels but apparently no corresponding change in protein abundance; examples include HSP70-2 or ornithine aminotransferase (see Figure 5 and Additional data file 2). These observations could be accounted for by the same explanations given for the cases of delayed degradation, except that here the proteins may be even more resistant to degradation and/or their transcript levels may have a still lower threshold at which translation already occurs at a maximum rate. In the case of greater resistance to protein degradation, one would expect to observe a drop also in protein abundance after some more time if transcript levels remained low. Similarly, for both T-complex protein 1 subunit α and EXP-2 (Additional data file 2) the microarray data suggest a sudden, approximately twofold reduction in transcript levels that is not mirrored in the concomitant protein abundance profiles. Apart from the possible explanations mentioned above, here the apparently short duration of these transcript level changes might be 'smoothed out' by the potentially more slowly reacting protein abundance.

In contrast, the abundance profiles for a number of proteins/isoforms (M1-family aminopeptidase, HSP70-1, hydroxyethylthiazole kinase, proteasome component C8, triose phosphate isomerase, and uridine phosphorylase; see Figure 5 and Additional data file 2) show transcript level increases toward the end of the time course that are not registered in the protein abundance measurements. This may indicate a simple lag between transcript and protein synthesis or some more specific translational repression [7,9]. It was shown previously that *Plasmodium *parasites can produce and 'store' transcripts in preparation for translation at a more appropriate, later time in the life cycle [7].

Even more surprising are the protein abundance profiles recorded for HSP60 (Additional data file 2). Although the transcript level exhibits a sustained decrease by approximately twofold, protein abundance increases. Possible explanations for this finding include the following: translational repression of the transcripts whose diminishing inhibitory activity later coincides with lower transcript levels; significant changes in protein turnover; and the presence of other HSP60 isoforms with different PTMs (see below) that have yet to be identified on the gel and are therefore not included in our analysis. Such additional isoforms could in fact make up the bulk of this protein in the cell, in which case the apparent protein abundance increase observed for the isoforms that we have identified as HSP60 would be due to an interconversion from other HSP60 isoforms.

### Protein isoforms and PTMs

One of the most striking results of this study is the insight into the abundance and regulation of *Plasmodium *protein isoforms. It is evident that many proteins occur *in vivo *in more than one isoform because of pretranslational events such as alternative splicing or transcription initiation/termination and because of PTMs such as phosphorylation, acetylation, ubiquitination, cysteine oxidation, and protein cleavage (see, for example, reference [33]). Most such modifications and shifts from one isoform to another are invisible to common transcript analyses as well as many conventional proteomics analyses. Thus, at the present time, very little is known about their role in the *Plasmodium *life cycle.

In this study we identified five protein isoforms that correspond to the *P. falciparum *eIF4A, an RNA helicase with probable function in translation initiation that is essential for growth [34]. Three of these isoforms exhibit a protein expression pattern that is almost perfectly anticorrelated to that of two other isoforms corresponding to the same protein. It may be that the observed patterns correspond, at least in part, to the direct interconversion of protein isoforms 4 and 5 into isoforms 1 to 3, and back again. The nature of the PTMs giving rise to these five isoforms of eIF4A in *P. falciparum *has not been investigated, but the lateral shift on two-dimensional gels is consistent with phosphorylation of this protein, a modification previously observed in plants and yeast [52-55]. In plants the eIF4A phosphorylation state has been observed to change after diverse stimuli such as heat shock, hypoxia, and pollen tube germination, whereas in the *Plasmodium*-related parasite *Toxoplasma gondii *eIF4A shows strict stage-specific expression regulated at the transcriptional level [56]. Furthermore, in *Drosophila *eIF4A has been shown to regulate directly the ubiquitin-mediated degradation of a transcriptional regulator [57]. Whether the *P. falciparum *eIF4A exerts its influence at the level of protein translation initiation and/or whether it has an effect on the expression of other proteins by affecting ubiquitin-mediated degradation remains to be elucidated. Either way, the fact that its transcript levels cycle throughout the IDC [2] (Figure 5) and that its protein isoform states change during schizont development is consistent with an involvement in controlling developmentally regulated protein expression in this parasite.

Actin-I was also identified as five isoforms in this study. One of these (spot 5, Figure 1) appears truncated, considerably shifted toward a more alkaline pI, and it exhibits a drastically different expression profile compared with the other four isoforms (Figure 5). Their parallel arrangement on the gel (Figure 1) is consistent with these isoforms differing in their phosphorylation status, although we note that phosphorylation of actin has been observed in mammals, plants, and slime molds [58-62]. Similarly, we show the glycolytic enzyme enolase to be present in at least seven isoforms (Figure 6a), including two truncated versions (spots 3a and 4a) that apparently lack their amino-terminus, as judged by the peptide coverage revealed during the mass spectrometric analysis (data not shown). Whether the shortened isoforms of actin and enolase represent mere degradation byproducts or actually serve specific functions is an interesting open question. It is currently also unknown whether they originate from proteolytic cleavage or alternative pretranslational events. In *P. yoelii *some PTMs of enolase have been shown to be due to phosphorylation, with different isoforms being localized in different cellular compartments [21]. In the related parasite *T. gondii *two isoenzymes (encoded by two separate genes) show substantial localization in the nucleus and are expressed in a stage-specific manner [63]. Enolase has been reported to serve a number of functions unrelated to glycolysis in various organisms (see, for example, the references list in the report by Ferguson and coworkers [63]), and in the Apicomplexa it has been suggested to be involved in host cell invasion by *Plasmodium *and *Eimeria *and in the regulation of stage-specific gene transcription in *Toxoplasma *[63-65]. Interestingly, aldolase - another glycolytic enzyme - was previously shown to be part of the molecular invasion machinery of apicomplexan parasites [66]. Its possible involvement, in host cell invasion and gene regulation, its high absolute expression level (the enolase protein spots are among the most intense on silver stained two-dimensional gels), and its multiple protein isoforms therefore make enolase a highly intriguing protein that clearly merits further study.

## Conclusion

Our direct comparisons of relative transcript and protein abundance levels uncover a dynamic and complex picture of stage-specific gene and protein expression in *P. falciparum*. Particularly revealing are the insights into differentially expressed isoforms of some proteins that offer a glimpse of an almost bewildering complexity that may lie beneath corresponding RNA profiles and a deceptively simple appearance of overall protein abundance. Many of these isoform changes are fundamentally undetectable in transcript-level analyses and are invisible even to common protein-level investigations such as Western blotting and conventional high-throughput proteomics based on mass spectrometry. Our data reveal significant and distinct isoform changes for several proteins (for example, eIF4A, eIF5A, and HSP70-2) as the malaria parasites progress through the late stage of their intra-erythrocytic life cycle. The high reproducibility and temporal specificity of our observations strongly suggest that these changes are more than inconsequential fluctuations and that they represent biologically significant modifications. It is likely that many of these isoforms lead to different biological functionality of the cognate protein.

In the future, extending DIGE-based proteome profiling beyond the 12-hour schizont-stage window analyzed here to cover the entire intra-erythrocytic life cycle of *P. falciparum *(and also beyond the pH 3 to pH 7 isoelectric focusing [IEF] range) will lay the foundation for the proteomic exploration of the parasites' response to inhibitors or the differences between diverse strains from time course and protein isoform oriented perspectives.

## Materials and methods

### Cell culture and parasite sampling

*P. falciparum *parasites were initially grown in flasks under standard conditions [67]. In short, washed human red blood cells (RBCs) were kept at 2% parasitemia in RPMI 1640 medium, including 25 mmol/l HEPES (GIBCO, Life Technologies, San Diego, CA, USA) supplemented with 0.25% AlbuMAX II (GIBCO), 2 g/l sodium bicarbonate (Sigma, St. Louis, MO, USA), 0.1 mmol/l hypoxanthine (Sigma), and 10 mg/l gentamycin (GIBCO), at 37°C, 5% carbon dioxide, and 3% oxygen. Parasites were synchronized by sorbitol treatments (5% sorbitol for 10 minutes at room temperature) over several generations at 5 hours and/or 20 hours after the start of RBC invasion. A tightly synchronized culture was transferred at the beginning of RBC invasion to a Labfors bioreactor (Infors, Bottmingen, Switzerland). After 5 hours of invasion at 14.7% hematocrit in 'bioreactor medium' (containing a total of 0.5% AlbuMAX II and 37.5 mmol/l HEPES at pH 7.4), the culture was diluted to 1% hemotacrit. After intra-erythrocytic growth over 42 hours, including regular medium replacement, a second round of RBC invasion at high hematocrit was allowed to take place over 6 hours. The correct progression of the resulting culture was monitored every 2 hours for a total of 50 hours, and TP samples corresponding to 34, 38, 42, and 46 HPI were collected for further analysis. Parasitized RBCs were washed with phosphate-buffered saline (PBS) and lysed in 0.1% saponin (weight/vol in PBS) over 5 minutes at room temperature. Parasites were thoroughly washed with chilled PBS, pelleted, snap-frozen in liquid nitrogen, and stored at -80°C.

### Protein preparations

Lysis buffer (30 mmol/l Tris, 8 mol/l urea, 2 mol/l thiourea, and 4% CHAPS [pH 8.0] at room temperature) was added to the frozen parasite pellets, and the cells were disrupted by three cycles of freezing/thawing followed by sonication on ice over 10 minutes at 25% amplitude (with pulses of 2 seconds on, 3 seconds off, resulting in 4 minutes total pulse-on time). Insoluble material was pelleted for 30 minutes at 16,100 *g *at 4°C, followed by ultracentrifugation for 30 minutes at approximately 100,000 *g *at 4°C (TLA-120.1 rotor in an Optima Max ultracentrifuge; Beckman Coulter, Fullerton, CA, USA). Proteins in the cleared lysate were purified by chloroform/methanol precipitation, the pellet air dried, and the precipitated proteins resuspended in lysis buffer. Protein concentrations for all protein preparations were determined using the 2D-Quant Kit from Amersham (GE Healthcare Bio-Sciences AB, Uppsala, Sweden).

### DIGE labeling

For CyDye (GE Healthcare) minimal labeling, an aliquot of each protein preparation was divided into two parts, of which one half was labeled with Cy3 and the other half with Cy5 DIGE Minimal Dye Fluors (GE Healthcare; dissolved in anhydrous *N*,*N*-dimethyl-formamide) on ice for 30 minutes in the dark, using 0.4 nmol CyDye per 50 μg protein. A protein reference pool/internal standard consisting of equal amounts of the four TP samples was similarly labeled with Cy2. All labeling reactions were stopped by addition of 1 μl 10 mmol/l lysine per 0.4 nmol CyDye.

### Two-dimensional gels: first dimension

The first dimension of the protein separation (IEF) was performed using Immobiline DryStrips (GE Healthcare). For DIGE analysis 24 cm strips (pH3-7NL, nonlinear pH gradient) were loaded with 50 μg protein per CyDye (a total of 150 μg protein per strip) during rehydration, whereas for preparative silver-stained gels each 24 cm strip was loaded with 500 μg protein. To each protein sample to be loaded on a strip, immobilized pH gradient (IPG) buffer (pH 4 to 7; GE Healthcare) was added to a final concentration of 0.5%, and the total volume of the sample was adjusted to 450 μl by adding DeStreak Solution (GE Healthcare). Rehydration was carried out overnight in strip holders (GE Healthcare) placed in the Ettan IPGphor 3 instrument (GE Healthcare); a low voltage (30 V) was applied during the last 7 hours. IEF was typically carried out in the Ettan IPGphor 3 by ramping from 30 V to 1 kV over 1 kV hour (kVh), holding at 1 kV for 2 kVh, ramping from 1 kV to 8 kV over 13.5 kVh, and holding at 8 kV for 52 kVh, yielding a total of about 69 kVh.

For two-dimensional Western blots, IEF was carried out using 13 cm Immobiline DryStrips (pH 4 to 7) as described above but with the following modifications: each strip was loaded by rehydration with 500 μg protein in a total volume of 250 μl, and the IEF protocol consisted of ramping from 30 V to 1 kV over 1 kVh, ramping from 1 kV to 8 kV over 11.25 kVh, and holding at 8 kV for 12 kVh, yielding a total of about 24 kVh.

### Two-dimensional gels: second dimension

IPG strips were equilibrated under constant agitation at room temperature in the dark, first for 15 minutes in equilibration buffer (75 mmol/l Tris [pH 8.8], 6 mol/l urea, 30% glycerol, 2% SDS) supplemented with 1% (weight/vol) DTT (dithiothreitol), followed by 15 minutes in equilibration buffer supplemented with 2.5% (weight/vol) iodoacetamide. Strips were briefly washed in 1% SDS, placed atop an 11% polyacrylamide gel (25.5 × 20 cm), and overlayed with about 2 ml of a melted agarose solution (1× running buffer, 0.5% to 1% agarose, bromophenol blue). Second-dimension protein separation was achieved by running the gels in SDS buffer in an Ettan Daltsix Electrophoresis System (GE Healthcare). For 2D-DIGE using 24 cm IEF strips, proteins were run until the dyefront reached the bottom of the gel, whereas for two-dimensional Western blots using 13 cm IEF strips, the dye front was run only about 10 cm into the gel.

### DIGE data acquisition

Gels with CyDye-labeled samples were scanned on a Typhoon Trio scanner (GE Healthcare) at 100 μm resolution. The Cy3 channel was scanned with medium and the other two channels with normal sensitivity. The scanned images were cropped and imported into DeCyder 2D software, version 6.5 (GE Healthcare). After spot detection in the DIA module (differential in-gel analysis), spots were automatically matched across gels in the BVA (biological variation analysis) module. Spot assignments across all gels were then manually inspected, and where necessary spots were either re-matched or excluded from the subsequent analysis.

### DIGE data analysis

To arrive at relative protein abundance measurements using 2D-DIGE, we used the DeCyder 2D software, which first calculates the raw background-subtracted volume for each spot on a gel, which corresponds to the volume underneath its three-dimensional representation (see Figure 2a). Spot volumes observed in the Cy3 and Cy5 channels were normalized against the corresponding spot volumes observed in the Cy2 channel. To do so, two normalization factors are determined (one for the comparison Cy3/Cy2, and one for Cy5/Cy2), and all spot volumes observed in these channels (Cy3 or Cy5) are multiplied by the corresponding normalization factor.

The 'normalized volume ratio' values and one-way ANOVA *P *values were exported from DeCyder. For a given spot, the 'normalized volume ratio' refers to the ratio of the normalized spot volume observed in the Cy3 or Cy5 channel divided by the spot volume of this spot measured in the Cy2 channel, with Cy2 having been used exclusively to label the protein reference pool. By default, DeCyder annotates these volume ratios such that an x-fold increase and decrease in protein abundance is denoted by +x and -x, respectively (for instance, a 2-fold decrease being represented by -2). Thus, to calculate the standard deviation for the protein abundance values (volume ratios) for a given protein spot and TP (determined from four gels), the volume ratios were transformed such that an x-fold decrease in protein abundance is represented by 1/x (for example, a 2-fold decrease being represented by 0.5). The 'relative standard deviation' for each protein spot and TP was determined by dividing the standard deviation of its protein abundance values (volume ratios) by the arithmetic mean of those same values. The median of the four individual relative standard deviations across the four TP measurements was then taken as a measure of experimental reproducibility for a protein spot.

Finally, the volume ratios were log_2 _transformed such that an x-fold increase and decrease in protein abundance is denoted by +log_2_(x) and -log_2_(x), respectively (for example, a 2-fold decrease being represented by -1). For a given protein spot and TP, such log_2_-transformed volume ratios were determined from four gels, and averaging these values yielded that protein spot's average protein abundance at that TP. The averaged protein abundance values for all four TPs were then used to construct the abundance profile for that protein.

To classify the direction of change ('up', 'up-down', and so on) for protein expression profiles that showed significant change (ANOVA *P *< 0.01 and fold change >1.4×; see Figure 3b), we carried out pair-wise comparisons of abundance measurements (not log-transformed values) from neighbouring TP samples. To smooth the classification and disregard very small changes between neighbouring time point samples TP_n _and TP_n+1_, we only considered changes for which AbsoluteValue(Abundance [TP_n_] - Abundance [TP_n+1_])/Average(Abundance [TP_n_]; Abundance [TP_n+1_]) > 12.5%.

### Silver staining

Gels were fixed in a solution of 40% ethanol and 10% acetic acid, washed, and sensitized in 30% ethanol, 6.8% sodium acetate, and 0.2% sodium thiosulfate. After further washing, the gels were incubated in 0.25% silver nitrate for 15 to 20 minutes, washed, and developed in 2.5% sodium carbonate and 0.015% formaldehyde. The staining reaction was stopped in 1.5% Na_2_-EDTA.

### Two-dimensional gel spot identification by MS/MS

Protein spots were excised from preparative gels and destained in 15 mmol/l K_3_Fe(CN)_6 _and 50 mmol/l Na_2_S_2_O_3_, followed by washes in 100 mmol/l NH_4_HCO_3_, in 50 mmol/l NH_4_HCO_3 _in 50% (vol/vol) acetonitrile, and in acetonitrile. The destained gel pieces were dried in a vacuum centrifuge and digested overnight at 37°C in 12.5 ng/μl mass-spec grade trypsin Gold (Promega, Madison, WI, USA) in 50 mmol/l NH_4_HCO_3_. After collection of the supernatant, the digested peptides were further extracted from the gel with 20 mmol/l NH_4_HCO_3 _and with 5% formic acid/50% acetonitrile. The combined supernatants were purified and concentrated in 0.5% formic acid/acetonitrile and in acetonitrile using ZipTips (Millipore, Billerica, MA, USA). The peptides were resuspended in 0.1% (vol/vol) trifluoroacetic acid in 50% acetonitrile and spotted on a MALDI target plate together with matrix solution (saturated solution of a-cyano-4-hydroxycinnamic acid [CHCA] in 0.1% trifluoroacetic acid/50% acetonitrile). Peptide mass fingerprints and MS/MS fragment ion masses were generated by MALDI-TOF-TOF mass spectrometry (Matrix-Assisted Laser Desorption/Ionization Time-Of-Flight tandem mass spectrometry) at the proteomics core centers of Nanyang Technological University and the National University of Singapore. The peptide and ion masses were then used to query both general (GenBank nonredundant) as well as custom-made protein databases (including both *Plasmodium *sequences from PlasmoDB-5.3 [68] and human proteins) using Mascot Server software, version 2.2.01 (Matrix Science, London, UK). Search parameters were set as follows: enzyme specificity set to trypsin, allowing up to one missed cleavage, fixed modification to carbamidomethyl (C), variable modification to oxidation (M), peptide tolerance to ± 100 ppm, and MS/MS tolerance to ± 1 Da.

### Western blots

For one-dimensional Western blots, 10 μg protein were loaded per lane and separated on approximately 10 cm × 7 cm SDS-PAGE gels, whereas for two-dimensional blots 500 μg protein were loaded per IEF strip/gel (see above). PAGE-separated proteins were transferred to nitrocellulose membranes and visualized using the MemCode Reversible Protein Stain Kit (Pierce/Thermo Fisher Scientific, Waltham, MA, USA). The membranes were blocked for 1 to 1.5 hours in 3% to 4% skim milk powder (BioRad, Hercules, CA, USA) and 0.5% to 1% Tween20 in Tris-buffered saline (blocking buffer). Proteins were detected using antibodies (see Acknowledgements, below) at 1:500 to 1:2,000 dilutions in blocking buffer. Secondary antibodies conjugated to horseradish peroxidase (GE Healthcare) were employed at 1:2,000 to 1:4,000 dilutions and visualized using SuperSignal chemiluminescent substrates (Pierce) or ECL kit (GE Healthcare) and X-ray film (Kodak).

### Microarray analysis

Relative RNA abundance levels were determined based on a set of saponin-lysed parasite pellets identical to those used for protein analysis by 2D-DIGE. RNA extraction, cDNA synthesis and labeling, as well as microarray hybridizations of the four samples representing time points 1, 2, 3, and 4 against a reference RNA pool were carried out as described previously [2]. Hybridizations were performed for 16 hours at 65°C using a Maui hybridization system (BioMicro Systems, Salt Lake City, UT, USA). Microarray data were aquired with GenePix Pro 6.0 software (Axon Instruments, Union City, CA, USA), and Lowess normalization and subsequent filtering for quality control were carried out using Acuity 4.0 (Axon Instruments). Spots were considered to be of good quality when they were unflagged and had a median intensity greater than the local background plus 2 times the standard deviation of the background for each dye channel. The complete microarray data are available at the Gene Expression Omnibus database at the National Center for Biotechnology Information [GEO:GSE13251].

## Abbreviations

ANOVA: analysis of variance; 2D-DIGE: two-dimensional differential gel electrophoresis; eIF: eukaryotic initiation factor; HPI: hours post-invasion; HSP: heat shock protein; IDC: intra-erythrocytic developmental cycle; IEF: isoelectric focusing; IPG: immobilized pH gradient; kVh: kV hour; MS/MS: tandem mass spectrometry; PBS: phosphate-buffered saline; pI: isoelectric point; PTM: post-translational modification; RBC: red blood cell; TP: time point.

## Authors' contributions

BJF designed experiments, carried out cell culture, set up two-dimensional gel protocols, analyzed data, and wrote the manuscript. NZ ran two-dimensional gels, prepared samples for mass spectrometry, and carried out Western blots. SM performed microarray experiments. PRP conceived of the study. ZB conceived of and supervised the study, designed experiments, and participated in writing the manuscript. All authors read and approved the final manuscript.

## Additional data files

The following additional data are available with the online version of this paper: a figure illustrating the quantitative 2D-DIGE raw data (Additional data file 1); a figure comparing relative mRNA and protein abundance expression profiles (Additional data file 2); a table presenting microarray data for the 24 genes corresponding to the parasite proteins identified in this study (Additional data file 3); and a table listing the complete microarray raw data (Additional data file 4).

## Supplementary Material

Additional data file 1 Quantitative 2D-DIGE raw data. The panels show the individual raw data points for all protein isoforms identified in this study. The volume ratios have not been mean-centered around zero. Click here for file

Additional data file 2 Expression profiles comparing relative mRNA and protein abundance. See legend to Figure 5 in the main text. Click here for file

Additional data file 3 Microarray data for the 24 genes corresponding to the parasite proteins identified in this study. The transcript measurements represent normalized ratios of the timepoint samples versus a *P. falciparum *RNA pool and have been log_2_-transformed, averaged (in the case of multiple oligos per gene), and mean-centered around zero. Click here for file

Additional data file 4 Complete microarray raw data. The transcript measurements represent normalized ratios of the timepoint samples versus a *P. falciparum *RNA pool and have been log_2 _transformed. These microarray data are also available at the Gene Expression Omnibus data base at the National Center for Biotechnology Information [GEO:GSE13251]. Click here for file
