# Supplementary figures and images for: Quantitative protein expression profiling reveals extensive post-transcriptional regulation and post-translational modifications in schizont-stage malaria parasites
Source: Genome Biol. 2008 Dec 17;9(12):R177. doi: 10.1186/gb-2008-9-12-r177 (PMC2646281; doi:10.1186/gb-2008-9-12-r177)

## Additional file 1

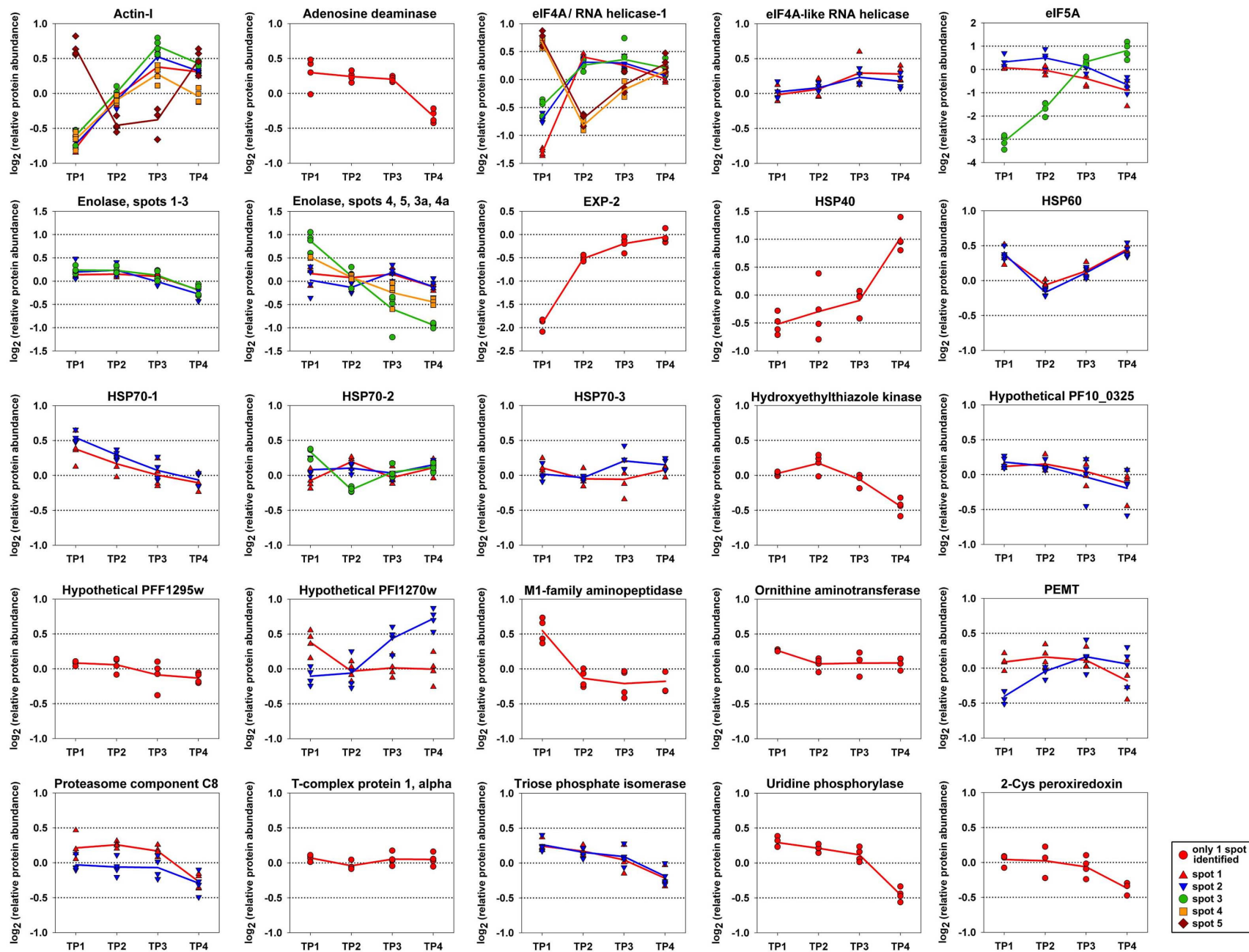

Supplement: Additional data file 1 — Quantitative 2D-DIGE raw data. The panels show the individual raw data points for all protein isoforms identified in this study. The volume ratios have not been mean-centered around zero. [file gb-2008-9-12-r177-S1.pdf]

## Additional file 2

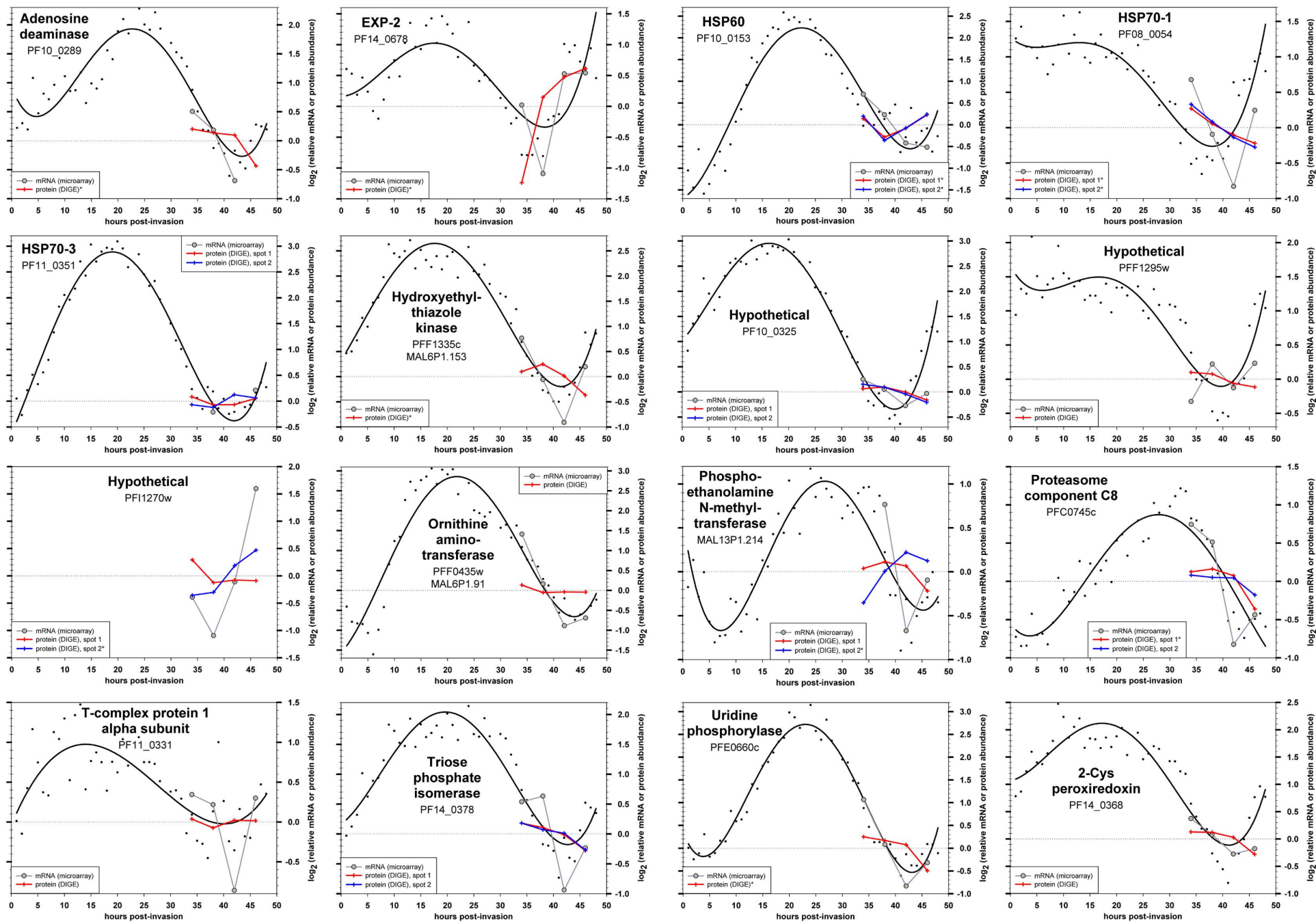

Supplement: Additional data file 2 — Expression profiles comparing relative mRNA and protein abundance. See legend to Figure 5 in the main text. [file gb-2008-9-12-r177-S2.pdf]
